# Fatality after deliberate ingestion of the pesticide rotenone: a case report

**DOI:** 10.1186/cc3528

**Published:** 2005-04-29

**Authors:** David Michael Wood, Hadi Alsahaf, Peter Streete, Paul Ivor Dargan, Alison Linda Jones

**Affiliations:** 1 Specialist Registrar in General Medicine and Clinical Pharmacology, Department of Pharmacology and Clinical Pharmacology, St George's Hospital Medical School, London, UK; 2 Consultant in Anaesthetics and Intensive Care Medicine, Kingston Hospital, Kingston, Surrey, UK; 3 Head of Clinical & Forensic Toxicology Section, Medical Toxicology Laboratory, Guy's and St. Thomas' NHS Foundation Trust, London, UK; 4 Consultant Clinical Toxicologist, National Poisons Information Service (London), Guy's and St. Thomas' NHS Foundation Trust, London, UK; 5 Director and Clinical Toxicologist, National Poisons Information Service (London), Guy's and St. Thomas' NHS Foundation Trust, London, UK

## Abstract

Rotenone is a pesticide derived from the roots of plants from the Leguminosae family. Poisoning following deliberate ingestion of these plant roots has commonly been reported in Papua New Guinea. However, poisoning with commercially available rotenone in humans has been reported only once previously following accidental ingestion in a 3.5-year-old child. Therefore, the optimal management of rotenone poisoning is not known. After deliberate ingestion of up to 200 ml of a commercially available 0.8% rotenone solution, a 47-year-old female on regular metformin presented with a reduced level of consciousness, metabolic acidosis and respiratory compromise. Metformin was not detected in premortem blood samples obtained. Despite intensive supportive management, admission to an intensive care unit, and empirical use of *N*-acetylcysteine and antioxidant therapy, she did not survive. Poisoning with rotenone is uncommon but is potentially fatal because this agent inhibits the mitochondrial respiratory chain. *In vitro *cell studies have shown that rotenone-induced toxicity is reduced by the use of *N*-acetylcysteine, antioxidants and potassium channel openers. However, no animal studies have been reported that confirm these findings, and there are no previous reports of attempted use of these agents in patients with acute rotenone-induced toxicity.

## Introduction

Rotenone is a botanical pesticide derived from the roots of species of plants from the family Leguminosae. Most commercially available preparations are derived from the species *Derris elliptica*, *Derris mallaccensis*, *Lonchocarpus utilis *and *Lonchocarpus urucu*. It has pesticide activity against a wide variety of insects and arachnids encountered in both domestic and commercial horticulture on bush and vine fruits, fruit trees, shade trees, flowers, shrubs and vegetables [1]. Ingestion of naturally occurring rotenone was previously commonly reported as a method of deliberate suicide in natives of New Ireland in Papua New Guinea, who were seen to eat the roots of plants known to contain rotenone prior to their death [2].

There has been only one other reported fatality, in a 3.5-year-old girl, following ingestion of commercially available rotenone solution [3]. Because the deliberate ingestion of commercially available rotenone in humans is uncommon, the optimal management of rotenone poisoning is not currently known. We report here a case of a fatality following ingestion of commercially available rotenone that did not respond to maximal supportive care and treatment with *N*-acetylcysteine (NAC) and other antioxidants.

## Case report

A 47-year-old woman weighing 64 kg and with known type 2 diabetes mellitus managed with metformin (500 mg three times daily) presented after she had ingested up to 200 ml from a bottle of 0.8% rotenone solution (Bio Liquid Derris Plus™; PBI Home & Garden Limited, Waltham Cross, UK). The maximum dose ingested would therefore have been 1.6 g, equating to 25 mg/kg. She was bought to the emergency department having been found collapsed and unconscious at home by her family, with a history of vomiting.

Her initial Glasgow Coma Scale (GCS) score was 3/15 and she was therefore intubated and ventilated; she had no requirement for sedative or paralyzing agents. After intubation, her blood pressure was 93/52 mmHg and heart rate 87 beats/min. Her electrocardiogram showed sinus rhythm. Baseline blood investigations showed normal renal function, but liver dysfunction with an elevated alanine transaminase of 233 IU/l. Arterial blood gases showed a severe metabolic acidosis (pH 7.09, arterial O_2 _tension 24 kPa, arterial CO_2 _tension 4.3 kPa, HCO_3 _10.3 mmol/l, base excess -19 and lactate 13 mmol/l). She was therefore commenced on continuous venovenous haemodialysis with lactate-free dialysate and infusion of 50 ml/hour sodium bicarbonate for management of her metabolic acidosis. Computed tomography scanning showed no intracranial abnormalities to explain her reduced GCS. She was transferred to the intensive care unit, and the National Poisons Information Service (London) was contacted for further advice on management.

A brown watery fluid (30 ml), similar to commercially available rotenone in smell and consistency, was noticed via nasogastric aspiration 6 hours after ingestion. Because previous *in vitro *studies have demonstrated a benefit of NAC and antioxidants in preventing rotenone toxicity in human cell lines [4-6], she was treated with intravenous NAC (standard Prescott protocol 1979) and other antioxidants (including multivitamins, 5 ml/day ketovite [orally] and 125 mg zinc sulphate three times daily [orally]) empirically. Also, 200 mg/day iron was administered intravenously; this has been shown to activate ATP-dependent potassium channels, which is protective in rotenone-induced toxicity [7].

She remained hypotensive despite fluid resuscitation, and her cardiac index on oesophageal Doppler studies was 7.7 l/min per m^2 ^with a stroke volume of 97 ml and systemic vascular resistance index of 470, indicating the presence of a vasodilated high output state. She was therefore commenced on a noradrenaline (norepinephrine) infusion in order to maintain her blood pressure, with a maximum dose 0.35 μg/kg per hour. Repeat cardiac studies using pulse contour cardiac output 12 hours after the initial presentation revealed a worsening cardiac index (2.5 l/min per m^2^), with a reduced stroke volume of 30 ml and an increased systemic vascular resistance index of 2359, indicating the presence of a vasoconstricted low output state. She was therefore started on dobutamine (maximum dose 10 μg/kg per h) and weaned from the noradrenaline infusion.

After initial stabilization of her clinical state with maximal supportive care, she then started to deteriorate clinically, with signs of cardiovascular collapse and no signs of obvious neurological recovery. At 48 hours after admission she suffered an asystolic cardiac arrest, which did not respond to cardiopulmonary resuscitation. At postmortem there were signs of multiorgan failure, with pulmonary oedema and congestion of the heart, spleen and kidneys. The liver was icteric with centrilobular necrosis and general disintegration.

## Results

Samples of serum were obtained at the time of admission and analyzed locally and by the Medical Toxicology Laboratory in London. There is currently no available method for the quantification of rotenone concentrations, and we were unable to identify a suitable technique for rotenone analysis. However, analysis of serum samples failed to detect the presence of other drugs, including drugs of abuse, alcohol, barbiturates, anticonvulsants, or tricyclic antidepressants. Importantly, there was no detectable metformin in the serum samples that could have accounted for the patient's lactic acidosis.

## Discussion

Poisoning with the plant-derived pesticide rotenone is uncommon and potentially fatal. In the case presented here, the patient presented after ingestion of up to 200 ml of a 0.8% (1.6 g) commercially available rotenone solution (Bio Liquid Derris Plus™) with a reduced GCS score and significant metabolic acidosis. Despite meticulous supportive care in the intensive care unit and treatment with NAC, and empirical use of antioxidants (oral multivitamins, oral zinc sulphate and intravenous iron), she did not survive.

Rotenone is a pesticide derived from the roots of members of the Leguminosae family of plants. The roots of these plants were used for many years by the Chinese because of their pesticidal actions [1]. It was first extracted from these plant roots in 1895 and was patented for use as a pesticide in the UK in 1912, although the chemical structure of rotenone was not determined until 1932. Rotenone has been demonstrated to have activity against a wide variety of insects and arachnids, and against vertebrate fishes [1,8]. Commercially available rotenone is limited mainly to domestic use because it rapidly decomposes when exposed to sunlight and the duration of its biological activity is approximately 1 week after use.

Authors have previously reported that ingestion of plant roots known to contain rotenone was common among individuals attempting suicide in the New Ireland region of Papua New Guinea [2]. Although no assays to confirm rotenone ingestion were available, individuals were often seen to have been eating the plant roots before their death, or the chewed roots would be found in close proximity to a deceased individual. The deliberate or accidental ingestion of commercially available rotenone is uncommon, and there has been only one other case report of a fatality, in a 3.5-year-old girl, following accidental ingestion of rotenone [3].

The toxicity of rotenone in animal studies is variable. The 50% lethal dose (LD_50_; i.e. the dose required to kill 50% of the population studied) varied from 13 to 130 mg/kg in guinea pigs [9-11] and from 25 to 132 mg/kg in rats [9,10,12,13] to 1500 mg/kg in rabbits [11]. In humans the minimum LD is not known, but death occurred in a 3.5-year-old child who had ingested 40 mg/kg rotenone solution [3]. Some of the differences seen in the fatal doses of rotenone may reflect differences in the preparations that were used or ingested, in addition to species differences in toxicity [14]. Higher doses are required for water-based than for fat-based preparations, because rotenone is very poorly soluble in water. Additionally, in animals given rotenone by subcutaneous, intravenous, or intraperitoneal routes, the LD_50 _was much lower and this probably reflects the rapid first pass metabolism of rotenone by the liver [11].

In animal studies, classical signs following acute ingestion of rotenone include initial respiratory stimulation, followed by significant respiratory depression and respiratory arrest [1]. Death occurs in the first 30 min in roughly half of animals given intraperitoneal rotenone [10] and within 2 days in animals that ingested oral rotenone [12]. Other features in animal studies of rotenone toxicity include vomiting, incoordination, convulsions and muscular tremors. Postmortem studies in the animals that died also demonstrated pulmonary congestion [10,15] and gastrointestinal irritation [10,11]. Individuals who were known to have ingested plant roots in New Ireland were reported to suffer from profound vomiting, dilated pupils and feeble pulse before death, and autopsies in fatal cases showed acute congestive heart failure [2]. In the previous reported accidental overdose, the child suffered from vomiting, severe metabolic acidosis (pH 6.76), drowsiness, coma and respiratory depression leading to respiratory arrest [3]. Following death, postmortem studies showed anoxic damage to the brain, lungs and heart, with an associated sero-haemorrhagic pleural effusion, acute tubular necrosis and significant gastrointestinal irritation and haemorrhage. In the case reported here, the patient presented to hospital with a significant reduction in level of consciousness, associated respiratory depression and severe metabolic acidosis, and at postmortem there were signs of multiorgan failure and significant liver damage.

The toxicity of rotenone has been more widely investigated in neuroblastoma cell lines and rotenone-induced animal models of Parkinson's disease. In rats given systemic rotenone for up to 20 days, 80% exhibited systemic toxicity with autopsy findings of severe liver necrosis [16]. In models of acute toxicity, rotenone was shown to cause both dose and time dependent reductions in neuroblastoma cell line viability [4,17-19]. Mortality in rats given subcutaneous rotenone was proportional to the doses administered, with 0% mortality with 10 mg/kg increasing to 40% mortality with 15 mg/kg [20]. Rotenone is known to be a potent inhibitor of complex I of the mitochondrial respiratory chain in all cell types, by inhibiting the function of mitochondrial NADPH (nicotinamide adenine dinucleotide phosphate, reduced form) dehydrogenase activity [5,6], therefore leading to a decrease in aerobic metabolism and development of a lactic acidosis. This inhibition of the mitochondrial respiratory chain leads to an increase in the production of hydrogen peroxide, and superoxide and oxygen radical species [6,21]. It is thought that the production of these oxidant species is the mechanism by which rotenone exerts its acute toxic effects, leading to fragmentation of DNA [6] and lipid peroxidation [6], increased lactate dehydrogenase release [4] and an increase protein carbonyl concentration, which is a marker of apoptotic cell death [5].

Inhibition of NADPH dehydrogenase within cells leads to a deficiency in the conversion of oxidized NADP^+ ^to reduced NADPH. This NADPH then is utilized by glutathione reductase to act as an electron donor to convert oxidized glutathione to reduced glutathione. Reduced glutathione acts within cells as an antioxidant, reducing cellular damage caused by oxidant molecules. In acute toxicity studies in the SH-SY5Y neuroblastoma cell line, rotenone concentrations of 5 μmol/l resulted in significant increases in oxidized glutathione concentrations and reductions in reduced glutathione concentrations [6]. Pretreatment of neuroblastoma cell lines with 100 μmol/l NAC prior to rotenone exposure resulted in decreased markers of oxidant stress, and levels of production of both hydrogen peroxide and superoxide oxygen radicals were reduced by approximately 80% compared with those cells exposed to rotenone alone [6]. Similarly, with pretreatment with NAC before rotenone exposure, there was reduction in DNA fragmentation, lactate dehydrogenase release and other markers of apoptotic cell death [4,6]. Additionally, overall neuroblastoma cell death was significantly reduced by pretreatment with 100 μmol/l and 500 μmol/l NAC prior to rotenone exposure [4,6].

As well as the glutathione hypothesis for hepatotoxicity in rotenone poisoning, in any patient who is hypotensive for a period of several hours, the possibility that this hypotension has caused 'shock liver' must be considered. Shocked liver is best avoided by maintenance of an adequate mean arterial blood pressure, but any hypotension should be controlled as aggressively and as soon as possible, and oxygenation maximized for recovery to take place. Initial oesophageal Doppler studies in this patient indicated a high cardiac index and vasodilatation. Despite subsequent administration of NAC (a hyperosmolar solution that is associated with vasodilatation and increased cardiac index [22]), the patient's cardiac index, as measured by pulse contour cardiac output, fell and she became vasoconstricted. This was a very poor prognostic sign indeed.

In addition to the previous studies using NAC to replenish glutathione, other antioxidants have been tested in an attempt to prevent rotenone-induced toxicity. In the neuroblastoma cell line SH-SY5Y, preincubation with the plant flavanoid fraxetin produced comparable reductions in rotenone-induced cell death and other markers of rotenone-induced cellular damage to NAC [4,6]. Also, other antioxidants such as α-tocopherol and coenzyme Q_10 _have been shown to reduce rotenone-induced cell death [5]. Potassium channel opening drugs such as iptakalin [23] and diazoxide [24] also appear to reduce the toxicity of rotenone in cell models. It is proposed that potassium channel opening drugs cause prolonged hyperpolarization of cells, therefore leading to cellular protection. However, the exact mechanism of cellular protection is unclear, because some potassium channel opening drugs, such as glibenclamide, are only partly protective in preventing rotenone-induced toxicity [23]. Other drugs shown to reduce rotenone toxicity in neuroblastoma cell models include pranipexole [25] and prostaglandin A_1 _[19].

However, no studies have been conducted in whole animal models of rotenone-induced toxicity to confirm whether the use of NAC, antioxidants and potassium channel opening drugs reduce the toxicity of rotenone, as shown in neuroblastoma cell line studies. Additionally, there have been no other reported cases of attempted use of NAC or other potentially beneficial antioxidants in the management of acute rotenone exposure or toxicity in humans. This patient had established toxicity on presentation (coma, hypotension and severe metabolic acidosis), and so it is difficult to predict, based on this case, whether earlier intervention with agents such as NAC would have an impact on outcome.

## Conclusion

We describe here a case of a fatality following severe rotenone poisoning. The patient presented with a reduced GCS score and severe metabolic acidosis that did not respond to meticulous supportive care, treatment with NAC and empirical use of antioxidants. Other toxicological causes of the metabolic acidosis, for example metformin toxicity, were excluded. The optimal management of patients who present following rotenone ingestion is still not known, but future use of NAC and antioxidants shown in cellular models to reduce rotenone toxicity may help to improve survival, although their use is cautioned in patients with haemodynamic compromise.

## Key messages

• 	Reports of rotenone toxicity in humans are rare and consequently the optimal management of rotenone toxicity is not known.

• 	In vitro cell line studies have suggested that rotenone toxicity can be revented by the use of N-acteyl cysteine, anti-oxidants and potassium channel openers. There have been no animal studies to confirm these observations.

• 	In this reported case, the use of N-acteyl cysteine, antioxidants and potassium channel openers did not alter the outcome, although the patient had features of established toxicity on presentation.

• 	Future use of NAC and antioxidants may help to improve survival in patients with rotenone toxicity.

## Abbreviations

GCS = Glasgow Coma Scale; LD = lethal dose; NAC = *N*-acetylcysteine; NADPH = nicotinamide adenine dinucleotide phosphate, reduced form.

## Authors' contributions

HA was in charge of the patient's immediate care and management. DMW, PID and ALJ were involved in providing specialist advice concerning the management of this patient. PS undertook the serum drug and toxicological analyses. DMW was responsible for drafting the first draft of the manuscript and all authors read and approved the final manuscript.
